# Phenotypic and genotypic landscape of PROKR2 in neuroendocrine disorders

**DOI:** 10.3389/fendo.2023.1132787

**Published:** 2023-02-08

**Authors:** Julian Martinez-Mayer, Maria Ines Perez-Millan

**Affiliations:** Instituto de Biociencia, Biotecnología y Biología Traslacional (iB3), Departamento de Fisiología, Biología Molecular y Celular, Facultad de Ciencias Exactas y Naturales, Universidad de Buenos Aires, Buenos Aires, Argentina

**Keywords:** kallmann syndrome, hypogonadotropic hypogonadism, genetic diagnosis, pituitary, functional assay

## Abstract

Prokineticin receptor 2 (*PROKR2*) encodes for a G-protein-coupled receptor that can bind PROK1 and PROK2. Mice lacking *Prokr2* have been shown to present abnormal olfactory bulb formation as well as defects in GnRH neuron migration. Patients carrying mutations in *PROKR2* typically present hypogonadotropic hypogonadism, anosmia/hyposmia or Kallmann Syndrome. More recently variants in *PROKR2* have been linked to several other endocrine disorders. In particular, several patients with pituitary disorders have been reported, ranging from mild phenotypes, such as isolated growth hormone deficiency, to more severe ones, such as septo-optic dysplasia. Here we summarize the changing landscape of *PROKR2*-related disease, the variants reported to date, and discuss their origin, classification and functional assessment.

## Introduction

Prokineticin receptor 2 (*PROKR2*) encodes PROKR2, a 384 amino acids long G-protein-coupled receptor (GPCR). PROKR1 and PROKR2 share around 85% sequence identity, and share ligands, called prokineticins (PROK1 and PROK2). When the ligands bind to PROKR2, the GPCR activates several signaling pathways, including IP3/Ca2+ signaling, the MAPK pathway, and cAMP signaling. Prokineticin signaling is involved in different biological functions, including mood regulation, smooth muscle contraction, angiogenesis, neurogenesis, food intake and the circadian clock (1–6). PROKR2 shows localized distribution in the central nervous system, with particularly high expression in the suprachiasmatic nucleus and the olfactory bulbs (6, 7). *Prokr2^−/−^* knockout mice present abnormal olfactory bulb formation and severe atrophy of the reproductive system in both sexes, a phenotype similar to Kallmann Syndrome (KS) in humans (8). At the hypothalamic level, immunohistochemistry revealed a lack of neuroendocrine GnRH cells (gonadotropin-releasing hormone expressing neurons) (8). The same year Dodé et al. searched for mutations in *PROKR2* in patients that presented KS, linking for the first time *PROKR2* with pubertal delays and smell disorders (9).

Since then, the diminishing cost of massive parallel sequencing has allowed for more broad panels and recently, whole exome sequencing of patients not only with KS or hypogonadotropic hypogonadism (HH), but with other endocrine disorders as well. As a result of this expansion in diagnostic sequencing, *PROKR2* has been recently linked to pituitary diseases, such as isolated growth hormone deficiency (IGHD) (10–14) combined pituitary hormone deficiency (CPHD), pituitary stalk interruption syndrome (15, 16) and septo-optic-dysplasia (13, 17), as well as a rare case of central precocious puberty (18).

In light of this expansion in *PROKR2* phenotypes and number of patients sequenced in recent years, we aimed to collect *PROKR2* reported variants in clinical cases and to establish genotype-phenotype correlations where possible. We also performed an up-to-date ACMG re-classification of each variant, in association to all functional data available.

## Search strategy

**Figure 1:**
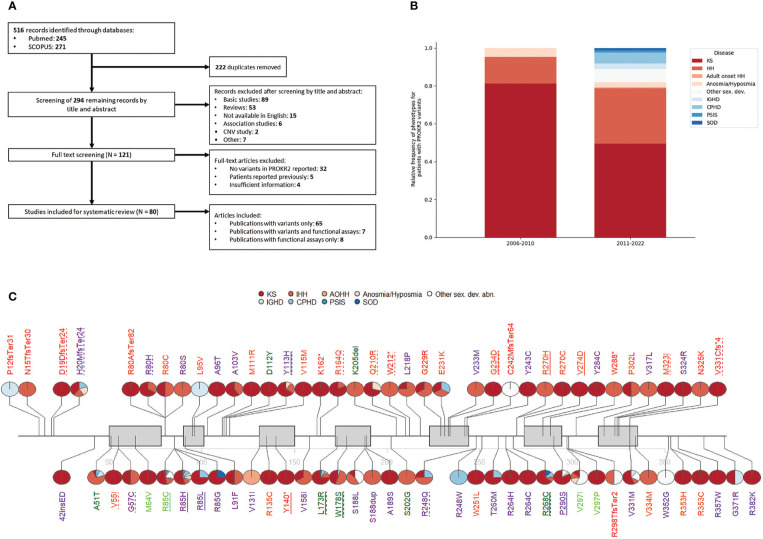
**(A)** Flow diagram showing the search and selection of studies. **(B)** Relative frequency of phenotypes in patients harboring *PROKR2* pathogenic or likely pathogenic variants. **(C)** Reported *PROKR2* variants. Pie charts next to each variant denote the percentage of patients carrying that variant that presented each phenotype. Each variant is coloured according to ACMG classification, where P = red, LP = orange, VUS = purple, LB = light green, B = dark green. Dotted underline means the variant was reported at least once as compound heterozygous and a full underline that it was reported at least once as homozygous. Gray boxes indicate transmembrane domains.

A literature search was conducted to identify studies describing human genetic variants in *PROKR2*, using two databases: PubMed and SCOPUS. The complete search strategy is presented in **Figure 1A**. From the included literature, the following data items were collected: described patient variants, their frequency, variant type, associated pathology and country of origin of the patient. Studies were excluded if patients had been reported previously or if studies were not available in English. Studies were screened by title and abstract and potentially relevant studies were reviewed by full-text analysis. The literature search identified 294 publications after the removal of duplicates. The titles and abstracts of these publications were screened and assessed for eligibility, resulting in 121 full-text articles deemed to be included in the review. Of these, after evaluation of the full text of the publications, 41 studies were excluded. Ultimately, a total of 80 studies were included in the present review (**Figure 1A**).

## Complex phenotypes caused by genetic variants in *PROKR2*

The absolute number of cases with *PROKR2* variants in the included literature was 435, carrying a total of 453 variants, 78 of those variants being unique. KS carried the highest number of cases (236), followed by HH (108). Both diseases are highly related, as evidenced by the co-occurrence of both phenotypes in family trees (19) and their shared genetic etiology (20). Anosmia and hyposmia without pubertal delay have also been reported, accounting for a small number of cases (9, 21–24). As has been described in mice, impairment of PROKR2-mediated migration of neurons, particularly interneurons, to the olfactory bulb is sufficient to explain the anosmia or hyposmia phenotype (25). Similarly, in the hypothalamus, the neuronal migration defect can impair GnRH secretion resulting in hypogonadism (8). In recent years, reports associating *PROKR2* variants to hypopituitarism and related disorders have emerged. Until now, 37 cases harboring *PROKR2* mutations presented pituitary diseases: the majority were CPHD and IGHD, followed by septo-optic dysplasia and pituitary stalk interruption syndrome (**Figure 1B**). Given the lack of a clear molecular explanation for this novel spectrum of *PROKR2*-related disease, further study on the role of *PROKR2* on pituitary development and disease is needed.

To date, one case of central precocious puberty has been described by Fukami et al. A female patient carried an heterozygous frameshift mutation (p.C242fsTer64), predicted to be gain of function based on Ca^2+^ mobilization assays. A gain of function in PROKR2 could lead to an untimely activation of the GnRH neurons and ultimately lead to CPP (18). However, another study including 31 female patients with central precocious puberty aimed to further strengthen the link between *PROKR2* and CPP and no genotype-phenotype correlations were found (26). We believe the putative link deserves further study and should be taken into consideration in future precocious puberty studies.

It is important to note that the majority of patients present heterozygous *PROKR2* variants. A small number of variants have been found in homozygous fashion (≃7%), and an even smaller number of cases present compound heterozygous inheritance (≃3%). Several mutations have also been found in apparently unaffected individuals, which suggests either incomplete penetrance or a digenic/oligogenic mode of inheritance in heterozygous patients (27). An example of this, is a patient presenting KS and homozygous early stop codon variant p.Y140*. Both parents are heterozygous for the same variant and are asymptomatic (28). Another two cases were reported with gain of stop codon variants: p.K162* and p.W288*, both in heterozygosity in patients with KS (29, 30). This seemingly different mode of inheritance (autosomal recessive vs autosomal dominant) for similar pathogenic variants points to either incomplete penetrance or oligogenicity as an intrinsic part of *PROKR2*-related disease.

With regards to oligogenicity, up to 20% of *PROKR2* cases are reported together with variants in another gene: the most commonly associated genes are: *FGFR1*, *CHD7*, *ANOS1*, *PROK2*, *GNRHR* and *SEMA3A*. The first case of oligogenicity was reported by Cost-Barbosa et al., who identified a patient with KS and ligand/receptor (i.e. *PROK2*/*PROKR2*) mutations (31). Variants in these two genes affect the same pathway, a ligand that does not bind properly together with a receptor that already has impaired signaling, results in disease and explains genotype-phenotype correlation. A similar idea can explain *GNRHR* oligogenic cases. A weakened GnRH signaling due to *PROKR2* mutations is further amplified by a defective receptor signal.

*ANOS1* is involved in GnRH neuron migration, similar to *PROKR2* (32). Although their mechanisms of action are different, a weakened gene regulatory network could explain how mutations in both genes at the same time could trigger a more severe disruption in neuron migration. *FGFR1* plays a role in neurite extension of GnRH neurons (33). We know that despite a probable defect in GnRH neuron migration, patients carrying *PROKR2* variants might still be healthy. We speculate that in patients that also carry *FGFR1* mutations, the combination of migration and neurite formation defects leads to disease at an increased rate.

*CHD7* has been historically linked to CHARGE syndrome (34). Although mice models show evidence for its role in olfactory bulb formation (35), its role in GnRH neurons has not been elucidated yet. Interestingly, there is also evidence that *SEMA3A* might play a role in CHARGE syndrome. It was shown that reduced expression *CHD7* also leads to reduced expression of *SEMA3A* (36). Heterozygous variants in *SEMA3A* cause KS and HH. Recently, two cases were reported with LP variants in *SEMA3A*, one case with CPHD and pituitary stalk interrption syndrome, the other with CPHD and heart/skeletal abnormalities (37). Given that both genes are often reported in oligogenic cases together with *PROKR2*, this begs the question of whether there are also common regulatory pathways between *CHD7/SEMA3A* and *PROKR2* that might influence GnRH neurons.

Overall oligogenicity is probably an important player in *PROKR2*-related disease and could help explain at least part of the incompletely penetrant phenotypes typically seen in these patients.

## *PROKR2* variants around the world

Most studies were performed in Asia (25 studies), Europe (22 studies) and North America (14 studies). In particular, the USA and China together amounted to more than half of the total patients studied. The low number of sequencing studies in Africa and South America is concerning, considering both continents together represent over 20% of the world population. Studies performed in these continents were also restricted geographically, to either northern Africa or just Argentina and Brazil in the case of South America. Variants circulating in these populations are currently an unexploited resource that could help reclassify known variants, as well as allow for the discovery of novel ones.

Some variants have a clear regional predominance. The p.W178S variant seems to be most prevalent in continental Asia (mainly in China, although it has also been reported in South Korea and Japan) (10, 30, 38–47). Nevertheless the variant has also been described in the USA and France, in patients of undisclosed ethnicity (9, 29). The high presence of this variant in Asian patients as well as its relatively high frequency in the east Asian population on GnomAD (1/500), point to it being a regional polymorphism (27).

Other missense variants unique to Asia are p.G57C, p.A103V and p.Y113H (23, 29–31, 38, 41–44, 46–51). The variants p.G57C, p.A103V were found in two and five patients with KS/HH respectively and both are classified as variants of uncertain significance (VUS) with a frequency of 1/1000 in this population. The variant p.Y113H is reported in twenty patients with a big range of phenotypes (KS, HH, CPHD, IGHD, SOD) and even though bioinformatic predictions mark it as pathogenic and functional studies show loss of function (LoF) (**Table 1**), it has high frequency in Gnomad (1/700 in East Asia), leading to its VUS classification (**Figure 1C**). Taking into consideration the total number of patients included in this review and their absence in other populations in GnomAD, it seems likely that these variants are indeed local. Further research is needed to determine whether these variants are pathogenic with incomplete penetrance and whether there are foundational effects.

**Table 1:** Variants in PROKR2 and their functional assessment.

Variant	Domain	Expression levels	MAPK/ERK pathway (G_i/o_)	IP3/Ca2+ release pathway (G_q_)	cAMP pathway (G_s_)	Binding	Functional assessment
42insED	ECL1	= ^44^	= ^44^	↓^44^	= ^44^	NE	LB
A51T	ECL1	**=** ^14,21,57^	= ^21^ or ↓^57^	**=** ^14,57^ or ↓^21^	= ^21^ or ↓^57^	**=** ^14,57^	CI
**V55I**	TM1	= ^21^	= ^21^	↓^21^	↓^21^	NE	LoF
M64V	TM1	↑^21^	= ^21^	= ^21^	↓^21^	NE	LB
**R80C**	ICL1	↓^21,56,57^	↓^21,56^ or =^57^	↓^21,56^ or =^57^	↓^21,57^	↓^56^ or =^57^	LoF
R85C	ICL1	**=** ^21,56,57^	**=** ^21,31,57^ or ↓^56^	**=** ^21,56,57^ or ↓^31,60^	= ^57^ or ↓^21^	**=** ^56,57,60^	LB
**R85H**	ICL1	↓^21,57,60^ or **=** ^14,56^	↓^21,56,57^	↓^21,57,60^ or = ^56^	= ^57^ or ↓^21^	**=** ^56,57,60^ or ↓^14^	LoF
**R85L**	ICL1	↑^21^ or ↓^57^	↓^21^ or Ø ^57^	↓^21,57^	↓^21^ or Ø ^57^	↓^57^	LoF
**R85G**	ICL1	↓^17,21^	↓^17,21^	↓^17,21^	↓^21^	NE	LoF
M111R	TM2	= ^21^	↓^21^	↓^21^	↓^21^	NE	LoF
D112Y	ECL2	= ^21^	= ^21^	= ^21^	↓^21^	NE	LB
**Y113H**	ECL2	↓^21,31,57^	↓^21,31^ or Ø ^57^	↓^21,31,57^	↓^21^ or Ø ^57^	↓^57^	LoF
**V115M** **R117W**	ECL2 ECL2	= ^21^ or ↓^31,57^ ↓^57^	↓^21,31^ or Ø ^57^ NE	↓^21,31,57^ NE	↓^21^ or Ø ^57^ NE	NE NE	LoF LoF
**R135C**	TM3	↓^21^	↓^21^	↓^21^	↓^21^	NE	LoF
V158I	ICL2	= ^21^ or ↓^53^	↓^21^	= ^53^ or ↓^21^	= ^53^ or ↓^21^	NE	CI
**R164Q**	ICL2	**=** ^21,57,59^	↓^21,31,57^	↓^21,31,57,59,60^	= ^57^ or ↓^21^	= ^57^	LoF
**L173R**	TM4	↓^14,31,53,57,60^ or = ^21^	↓^31,21^ or Ø ^57^	↓^21,31.53,57,60^	↓^21,53^ or Ø ^57^	↓^14^	LoF
**W178S**	TM4	↓^21,31,57,60,64^	↓^31,21^ or Ø ^57^	↓^21,31,60^	↓^21^ or Ø ^57^	NE	LoF
**S188L**	TM4	= ^21^ or ↓^31,57^	↓^31,21^ or Ø ^57^	↓^21,31^	↓^21^ or Ø ^57^	NE	LoF
S202G	ECL3	= ^21^	↓^21^	= ^21^	= ^21^	NE	LB
**Q210R**	ECL3	**=** ^21,57^	↓^21^ or Ø ^57^	↑^21^ or ↓^60^	↓^21^ or Ø ^57^	↓^57,60^	LoF
L218P	ECL3	= ^44^	= ^44^	Ø ^44^	= ^44^	NE	LB
**G229R**	TM5	↓^44^	↓^44^	Ø ^44^	Ø ^44^	NE	LoF
**E231K**	TM5	↓^44^	↓^44^	Ø ^44^	Ø ^44^	NE	LoF
**G234D**	TM5	Ø^57,64^ or ↓^21^	↓^21^ or Ø ^57^	↓^21^	↓^21^ or Ø ^57^	NE	LoF
**C242fs**	TM5	NE	NE	↑^18^	NE	NE	GoF
R248Q	ICL3	= ^21^	= ^31^ or ↓^21^	= ^21^ or ↓^31^	↓^21^	NE	CI
**W251L**	ICL3	= ^21^ or ↓^57^	= ^21^ or Ø ^57^	↓^21,57^	↓^21,57^	↓^57^	LoF
**T260M**	ICL3	= ^21^ or ↓^53^	↓^21^	↓^21,53^	↓^21,53^	NE	LoF
**R268C**	ICL3	**=** ^21,57^ or ↓^53^	↓^21^ or Ø ^53^	↓^21,53,60^ or = ^57^	↓^21,53^ or = ^57^	= ^57^	LoF
**R270H**	ICL3	**=** ^21,44^	↓^21^ or Ø^44^	↓^21^	**=** ^21,44^	NE	LoF
**R270C**	ICL3	↓^21^	↓^21^	↓^21^	= ^21^	NE	LoF
**V274D**	TM6	↓^53,57,58^ or ↑^21^	Ø ^57,58^ or ↓^21^	Ø ^21,57,58^ or ↓^53^	↓^21,53^ or Ø ^57^	NE	LoF
**P290S**	TM6	= ^21^ or ↓^57,60^ orØ ^64^	↓^21^ or Ø ^57^	Ø ^57,60^ or ↓^21^	↓^21^ or Ø ^57^	NE	LoF
V297I	ECL4	= ^21^	= ^21^	= ^21^	↓^21^	NE	LB
V317L	TM7	= ^21^	= ^21^	= ^21^	= ^21^	NE	B
M323I	TM7	↑^21^ or ↓^57^	↓^21,57^	**=** ^21,57,60^	= ^57^ or ↓^21^	= ^57^	CI
**N325K**	TM7	= ^21^	↓^21^	↑^21^	↓^21^	NE	LoF
V331M	TM7	↓^53,57,60^ or = ^21^	= ^21^ or ↓^57^	↓^31,53,57,60,61^ or ↑^21^	= ^53,57^	= ^57^	CI
**V334M**	TM7	↑^53^ or ↓^21^	↓^21^	↓^21,53^	↓^21,53^	NE	LoF
**R353H**	ICL4	= ^44^	↓^44^	↓^44^	= ^44^	NE	LoF
R357W	ICL4	= ^57^	**=** ^21,57^ or ↑^31^	↓^21,31^ or = ^57^	= ^57^ or ↑^21^	NE	CI
G371R	ICL4	NE	= ^21^	= ^21^	= ^21^	NE	B

The affected domain is shown (ECL, extracellular domain; ICL, intracellular domain; TM, transmembrane domain). For each functional aspect tested, the following convention was used: ↑, Increased; ↓, Reduced; Ø, No function; =, Unchanged; NE, Not evaluated; B, Benign; LB, Likely Benign; LoF, Loss of function; GoF, Gain of function; CI, Conflicting interpretation. Numbers indicate each corresponding reference.

Another variant with an interesting regional pattern is p.P290S. This variant was originally reported by Dodé et al. in French patients with KS, but it has been found at an increased frequency in Maghrebian patients. France’s population also has a high population percentage of immigrants with maghrebian origins (52), meaning the variants found in french cohorts could be attributed to maghrebian immigration. Functional testing indicates the variant is a LoF (**Table 1**). Nevertheless the variant is currently classified as a VUS, given its high frequency in Gnomad (0.0004).

Several patients have been reported with heterozygous nonsense variants, either due to a premature stop or a frameshift that results in a truncated protein. Although these variants are deemed to be causative of the disease, the relatively high prevalence of p.H20MfsTer24 in the European and Latino/Admixed population is intriguing (27). A founder effect may be at play in the European population, given that patients with these mutations have only been reported in Italy and France (9, 53). The symptoms of these patients were quite diverse (**Figure 1C**), ranging from KS/HH to CPHD and anosmia. It is also interesting to note that transcripts harboring such variants would likely be subject to nonsense mediated decay (NMD). NMD can trigger genetic compensation through increased transcription of adapting genes (54). PROKR2 and PROKR1 share ligands and their distinct actions are thought to arise from specificity at the level of tissue expression more so than functional differences (55). Given their high homology degree, incomplete penetrance due to genetic compensation for this type of variants seems plausible and warrants further investigation.

## The importance of functional assays

*PROKR2* is able to activate different G-proteins common to GPCR receptors, namely Gαi/o, Gαq and Gαs. Each of these pathways can be linked to canonical signal transduction pathways: MAPK/ERK pathway (Gαi/o), IP3/Ca2+ release pathway (Gαq) and cAMP pathway (Gαs). Several PROKR2 functional testing methodologies have been applied for each different pathway.

To study MAPK/ERK signaling two assays have been used. The most commonly used is the Egr1-luciferase assay (21, 31, 56). In this assay cells are transfected with wilde-type and mutant *PROKR2* expression plasmids, together with luciferase expression vectors. In this case the plasmid carries binding sites for EGR1, a downstream effector of p-ERK. After 24 hours the cells are then stimulated with a range of PROK2 concentrations, to generate a dose-response curve. The second assay is very similar, but instead of luciferase the final outcome measured is p-ERK protein levels through Western Blot analysis (44, 57). Variants are then evaluated on the percentage of activation that they elicit compared to wilde-type.

The IP3/Ca2+ release pathway (Gαq) was measured in several ways. The most frequent assay was aequorin-based (17, 31, 44, 58, 59). This method also relies on transfection, where *PROKR2* WT and mutant expression plasmids are co-transfected with an aequorin expression plasmid. Upon posterior activation of PROKR2 with PROK2, aequorin is activated by calcium release, producing light at 470 nm as an output signal. Another frequently used method was the Fluo-4 calcium reporter method (57, 60, 61). In this case Fluo-4 is added to the transfected cells, where it permeates the membrane. Once PROKR2 is activated, the subsequent intracellular calcium release triggers light emission by Fluo-4 in the 500-525 nm range.

In contrast, there are not many widely adopted cAMP pathway functional assays. Luciferase based assays have been used as described before, making use of the pGlosensor-22F cAMP sensor (21, 44). In this assay an engineered fused Luciferase is used, which carries a cAMP binding domain, and only emits light after a conformational change induced by cAMP binding (62). Other alternative functional assays have also been used, such as the commercial cAMP HTRF assay or RIA (53, 57).

Localization and expression of the receptor has been evaluated for multiple variants, mostly through Western Blot and immunofluorescence. Specific ligand binding affinity has also been tested for some variants using radioligand binding assays (14, 56, 57, 60, 63). For all reported variants where functional testing was available we took every assay into consideration and summarized the results in **Table 1**.

We labeled each variant as Benign (B), Likely benign (LB), conflicting interpretation (CI) or gain/loss of function (GoF/LoF), according to a qualitative consensus among studies. We considered a variant to be LoF if at least two assays showed a defect, or one assay was proven to cause loF and was tested by at least two independent publications, without conflicting results. Variants with just one defective assay were considered Likely Benign (LB), while those with none were considered Benign (B). It should be emphasized that functional assay results are not synonymous with pathogenicity. A clear example of this are variants L173R and R268C, which should be considered Benign according to ACMG criteria, despite their seemingly impaired signaling. They both present relatively high frequency in the general population, as well as homozygous individuals in GnomAD, but they could be possible risk factors given their increased frequencies in individuals with disease (64).

Despite some contradictions, generally functional assays are of great relevance for classifying most variants. Examples of this are the variants that fall in the transmembrane domain five (TM5): p.G229R, p.E231K and p.G234D. The functionally available data points to all of them being LoF, and this evidence leads to their classification as Pathogenic (P) or Likely Pathogenic (LP) (**Figure 1C**; **Table 1**). Two further variants in that same domain, p.V233M and p.Y243C, lack functional evidence and currently remain VUS. Remarkably over three quarters of the remaining VUS also lack functional testing. This exemplifies just how critical functional testing is for *PROKR2* variant assessment and subsequent genetic counseling.

## Conclusion

Since the initial report from Dodé et al. in 2006, many studies have reported *PROKR2* genetic alterations in patients with neuroendocrine disorders. A vast majority of these patients have insufficiencies in the gonadal axis, commonly with hyposmia or anosmia, while a minority present diverse forms of hypopituitarism.

This review provides a comprehensive catalog of published *PROKR2* variants described to date. Our catalog of predicted pathogenic variants may serve as a guideline for future genetic studies. In summary, this review represents a useful resource for clinicians overseeing *PROKR2*-related diseases and will help scientists to focus on the missing gaps of the literature to further cement its role in diseases concerning the hypothalamus-pituitary axis.

## Author contributions

All authors planned and wrote the paper. JM-M designed the figures. All authors contributed to the article and approved the submitted version.
